# Gender and vocal production mode discrimination using the high frequencies for speech and singing

**DOI:** 10.3389/fpsyg.2014.01239

**Published:** 2014-10-30

**Authors:** Brian B. Monson, Andrew J. Lotto, Brad H. Story

**Affiliations:** ^1^Department of Pediatric Newborn Medicine, Brigham and Women’s Hospital, Harvard Medical SchoolBoston, MA, USA; ^2^Speech, Language, and Hearing Sciences, University of ArizonaTucson, AZ, USA

**Keywords:** speech perception, acoustics, singing, voice, high-frequency

## Abstract

Humans routinely produce acoustical energy at frequencies above 6 kHz during vocalization, but this frequency range is often not represented in communication devices and speech perception research. Recent advancements toward high-definition (HD) voice and extended bandwidth hearing aids have increased the interest in the high frequencies. The potential perceptual information provided by high-frequency energy (HFE) is not well characterized. We found that humans can accomplish tasks of gender discrimination and vocal production mode discrimination (speech vs. singing) when presented with acoustic stimuli containing only HFE at both amplified and normal levels. Performance in these tasks was robust in the presence of low-frequency masking noise. No substantial learning effect was observed. Listeners also were able to identify the sung and spoken text (excerpts from “The Star-Spangled Banner”) with very few exposures. These results add to the increasing evidence that the high frequencies provide at least redundant information about the vocal signal, suggesting that its representation in communication devices (e.g., cell phones, hearing aids, and cochlear implants) and speech/voice synthesizers could improve these devices and benefit normal-hearing and hearing-impaired listeners.

## INTRODUCTION

Human vocalizations produce acoustic energy at frequencies above 6 kHz, but the perceptual impact of the upper portion of the speech spectrum is often not considered in speech perception research. This trend is likely due to the facts that vocal energy drops off rapidly above 5–6 kHz and that low-frequency speech cues are sufficient for high intelligibility (French and Steinberg, 1947; Fletcher and Galt, 1950). Thus high-frequency energy [(HFE), defined here as energy in the 8- and 16-kHz octave bands (5.7–22 kHz)] has traditionally been assigned a limited perceptual role in speech sound quality (Olson, 1947; Moore and Tan, 2003) and singing voice quality (Monson et al., 2011; see also Monson et al., 2014). There is a growing body of evidence that non-qualitative perceptual information is provided by the high frequencies, including cues for speech source localization and intelligibility (reviewed in Monson et al., 2014). For example, Best et al. (2005) showed that low-pass filtering speech at 8 kHz caused a significant increase in errors in the localization of the speech source in the horizontal (sagittal) plane, though there was no significant effect on localization in the lateral plane.

Stelmachowicz et al. (2001, 2007) showed that low-pass filtering speech at 5 kHz negatively affects the perception of the voiceless fricatives, especially for children. The effects they observed were generally restricted to the phoneme /s/. Pittman (2008), however, showed that the learning rate of children learning nonsense words with phonetic content that approximated the distribution of phonemes in American English was significantly poorer for speech low-pass filtered at 4 kHz than for speech low-pass filtered at 9 kHz. Thus children, whose high-frequency audiometric thresholds are typically much better than adults (Stelmachowicz et al., 1989), might be at the greatest risk for detrimental effects of HFE deprivation.

Lippmann (1996) found that adding HFE to consonant-vowel-consonant (CVC) tokens that had been low-pass filtered at 800 Hz caused a large increase in accuracy of consonant identification in adult listeners. Scores jumped from 44.3% correct (low-pass filtered at 800 Hz) to 74.9% correct when speech energy beyond 8 kHz was added. Apoux and Bacon (2004) found that filtering out a high-frequency band (3.5–10 kHz) caused a significantly larger drop in consonant identification scores for CV and VC tokens in noise than filtering out low-frequency bands. Since this band included energy down to 3.5 kHz, however, HFE cannot be singled out as the only contributing factor.

These findings bear on many aspects of speech and voice perception, but particularly communication devices (e.g., cell phones) and augmentative hearing devices that are now attempting to represent this frequency range. For example, while standard telephony has been restricted to the frequency range below 4 kHz, so-called “wideband” telephony or “HD voice” is now being integrated in applications within digital communication and Internet protocol (Geiser, 2012; Pulakka et al., 2012). There is also potential benefit for the hearing impaired (Moore, 2012), especially in noisy listening conditions. The results are mixed on how beneficial HFE is to hearing impaired adults, and the reason for this is not clear (reviewed in Moore et al., 2008). Badri et al. (2011) showed, however, that elevated audiometric thresholds at frequencies beyond 8 kHz were characteristic of otherwise normal-hearing listeners who complained of and exhibited poor performance on speech intelligibility with background noise. Moore et al. (2010) found a small but significant increase in intelligibility scores by increasing the cutoff frequency of low-pass filtered male speech from 5 to 7.5 kHz for normal-hearing adults performing speech-in-noise tasks when target speech and noise maskers were spatially separated. Fullgrabe et al. (2010) reported that listeners claimed they could sometimes recognize words when presented with only bandpass filtered speech from 5 to 10 kHz, although these claims were not rigorously tested. Berlin (1982) examined a handful of case studies of individuals with poor hearing in the low-frequency region but relatively good hearing in the HFE range, and reported good comprehension and articulation in these individuals.

The goal of the current study was to further assess the potential perceptual information provided by the high frequencies for speech and singing. We presented listeners with stimuli that consisted of *only* HFE extracted from speech and singing (similar to the study of Fullgrabe et al., 2010), necessitating the use of HFE information to perform behavioral tasks. Listeners were asked to perform gender and production mode (speech vs. singing) discrimination of the HFE tokens. Secondarily, and building on the results from Fullgrabe et al. (2010), listeners were asked *post hoc* to identify the words and song in the tokens they heard, which were all excerpts from the lyrics to a familiar song.

## MATERIALS AND METHODS

### STIMULI

Anechoic recordings were made for 15 subjects (eight female) who were native speakers of American English with no reported history of a speech or voice disorder. All singer/talker subjects had at least 2 years of post-high school private voice training. Age ranged from 20 to 71 years (mean = 28.5). The recordings were sung and spoken versions of “The Star-Spangled Banner” (SSB). Subjects were recorded speaking the lyrics first, using a note card (if desired), and were instructed to recite the lyrics in a conversational tone, pausing if necessary to look at the note card, but otherwise holding the note card down to the side. Subjects then produced the sung version of SSB. Subjects were allowed to sing in a key of choice from keys of G, A, B, C, or D Major. Subjects were instructed to perform the song as if in a real performance, incorporating desired artistic liberties, and were allowed to record the song as many times as desired.

**FIGURE 1:**
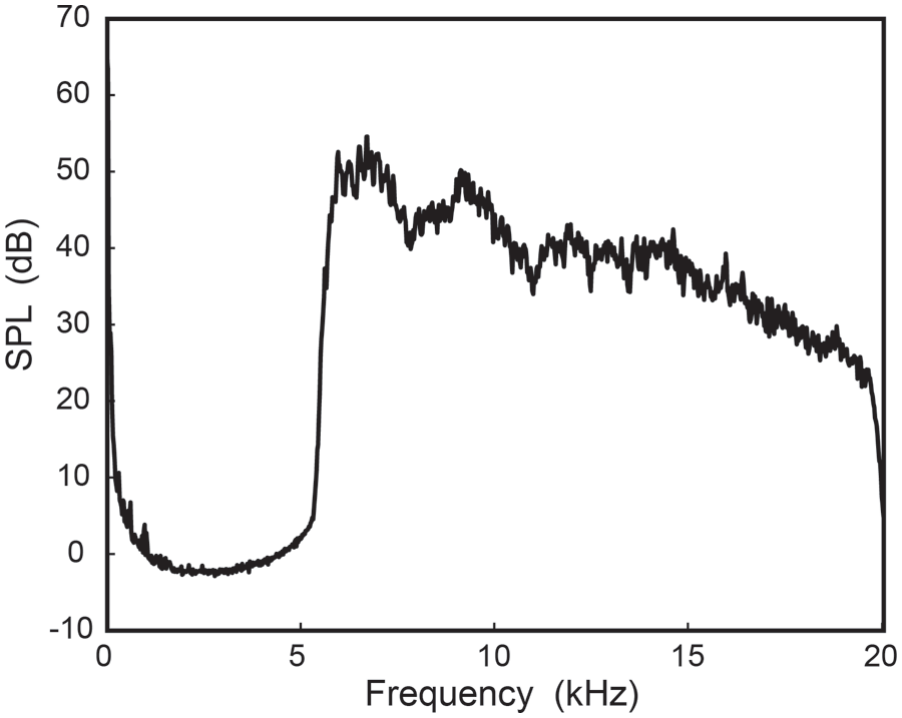
**Long-term average spectrum (LTAS) of high-frequency energy (HFE).** The HFE was extracted from the first 5 s of one male subject speaking the lyrics to the Star-Spangled Banner, recorded at the position of the listener (1 m from the loudspeaker, on axis).

Vocalizations were recorded at 24 bits and a sampling rate of 44.1 kHz using a precision condenser microphone located 60 cm directly in front of the mouth. (The recording apparatus and equipment are described in further technical detail in Monson et al., 2012). The SSB recordings were inspected and edited by hand to remove any unnatural pauses. Each recording was then passed through a digital Parks-McClellan equiripple FIR bandpass filter to extract HFE using cut-off frequencies of 5.7 and 20 kHz. Wave files were generated using the first and last 5 s of each SSB production mode by each subject. Generally, this time length resulted in the stimulus containing the phrases “Oh say, can you see” and “home of the brave” for singing; and “Oh say, can you see by the dawn’s early light what so proudly we hailed” and “banner yet wave o’er the land of the free and the home of the brave” for speech. A total of 60 stimuli were created (15 subjects × 2 segments × singing/speech). Stimuli were adjusted to have an overall level of 73 dB SPL. After this adjustment mean HFE octave band levels for the 8- and 16-kHz octave bands, respectively, were: 71 dB (standard deviation, SD = 0.4 dB) and 64.8 dB (SD = 1.7 dB) for female speech; 71.2 dB (SD = 0.3 dB) and 64.2 dB (SD = 1.3 dB) for female singing; 71.7 dB (SD = 0.1 dB) and 60.5 dB (SD = 2.1 dB) for male speech; and 71.6 dB (SD = 0.3 dB) and 60.8 dB (SD = 2.7 dB) for male singing. **Figure 1** shows the long-term average spectrum (LTAS) of the first 5 s of one male subject speaking the lyrics to the SSB, recorded in the listening environment setup (see Listening Conditions) at the position of the listener (i.e., this represents the actual signal spectrum presented to the listener).

### LISTENING CONDITIONS

The experiment took place in a standard double-walled sound booth with stimuli presented over a Mackie HR624 High Resolution Studio Monitor loudspeaker. The computer running the experiment was installed with a Lynx L22 sound card, with the output connected directly to the loudspeaker located in the booth. The frequency response of the loudspeaker and sound card had an on-axis response of ±5 dB from 100 to 20 kHz. Listeners sat in a desk directly in front of the loudspeaker, with the ear located a distance of 1 m from the loudspeaker. Listeners were asked to avoid large deviations from their sitting positions, and specifically not to lean forward toward the loudspeaker, but were not physically constrained. Stimuli were presented at an RMS level of 73 dB SPL. This level is approximately 25 dB higher than normal HFE levels in speech and singing (Monson et al., 2012).

### PARTICIPANTS

All listeners were recruited with informed consent as approved by the institutional review boards at the University of Arizona and Brigham Young University. Listeners received no compensation for their participation. Twenty-three listeners participated in the main experiment (14 female). Age ranged from 19 to 42 years, with a mean age of 24 years. Monaural audiometric thresholds were measured for all octave frequencies from 250 to 16 kHz with a GSI 61 Clinical Audiometer with high-frequency capability. Telephonics TDH-50P (294D200-2) headphones were used for regular audiometric frequencies (250–8 kHz), and Sennheiser HDA 200 headphones were used for high-frequency audiometry (8 and 16 kHz). This resulted in two thresholds obtained at 8 kHz for each ear. When these two thresholds differed, the better (lower) threshold was used. All listeners except one had thresholds better than or equal to 15 dB HL in at least one ear at all frequencies up to 8 kHz. One listener had thresholds of 30 and 20 dB HL at 4 and 8 kHz, respectively. Nine listeners had thresholds worse than 15 dB HL in both ears at 16 kHz. [Initially data for these listeners were analyzed separately, but since their scores did not differ significantly from the rest of the group (*t* = 0.771, *p* = 0.449) they were included in the results here.]

### PROCEDURE

The forced-choice perceptual task consisted of both a gender and production mode discrimination task, implemented with the Alvin software package (Hillenbrand and Gayvert, 2005). For each trial listeners were presented with one stimulus that they were to identify as one of four possible choices: Male Speech, Male Singing, Female Speech, or Female Singing. Following the initial presentation of the stimulus, listeners were allowed to repeat the stimulus presentation as many times as desired before giving a response, but were required to give a response before continuing to the next trial.

Responses were given by clicking on the desired on-screen button with a computer mouse, followed automatically by the presentation of the next trial. Listeners were given no feedback on the accuracy of their response. The total number of trials was 60 (one trial per stimulus). Stimulus presentation was randomized for each listener. The listening task lasted approximately 10 min. Listeners were given no indication prior to the listening task that they should attend to the song or words presented but were asked immediately following the experiment to identify in writing what song(s) the singers were singing and what the speakers were saying.

## RESULTS

**FIGURE 2:**
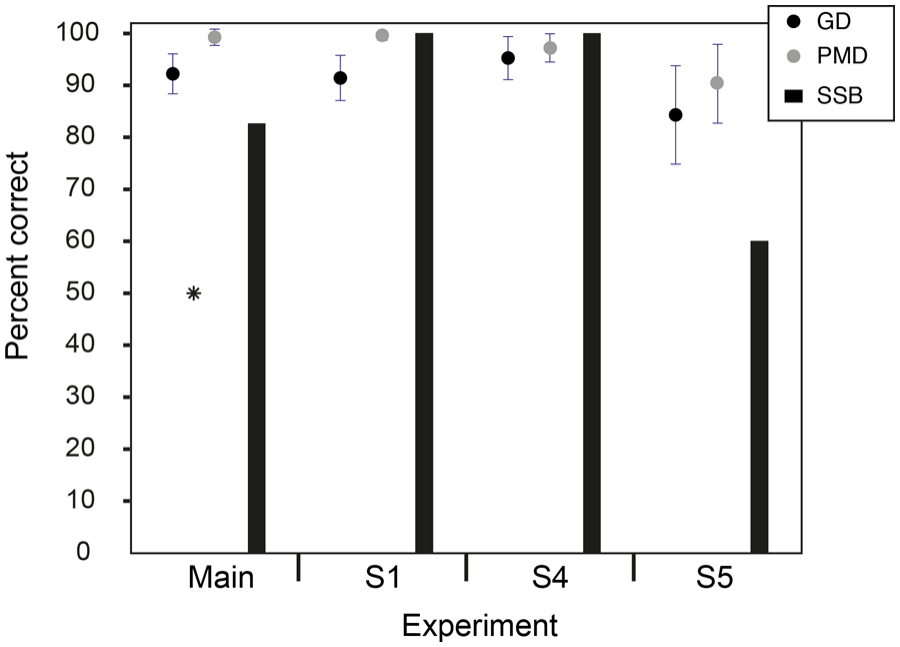
**Results of the main experiment and supplementary experiments.** Mean scores (percent correct) are shown for the gender discrimination (GD) and production mode discrimination (PMD) main experiment (no masker) and supplementary experiments S1 (with masker), S4 (decreased level), and S5 (speech levels). Scores were all well above chance (*). (Error bars are ± 1 SD). The percentages of listeners to correctly identify the SSB are also shown.

All listeners were able to perform the discrimination tasks successfully. **Figure 2** shows the mean scores (percent correct). Mean scores were 99.3% correct (SD = 1.6%) for production mode discrimination and 92.2% correct (SD = 3.8%) for gender discrimination. All listeners scored well above chance for both tasks (binomial test, *p* < 0.0001). All listeners but one correctly identified the song being sung as SSB. All listeners but three correctly identified the words being spoken as lyrics to SSB.

The difference in scores between production mode and gender discrimination was statistically significant (*t*= 8.388, *p* < 0.001). It was not surprising that listeners could more easily distinguish production mode than gender. One of the major differences between speech and singing is the duration of syllables and words. This temporal information is preserved in HFE. It was not expected, however, that listeners would perform so well on the gender discrimination task.

**FIGURE 3:**
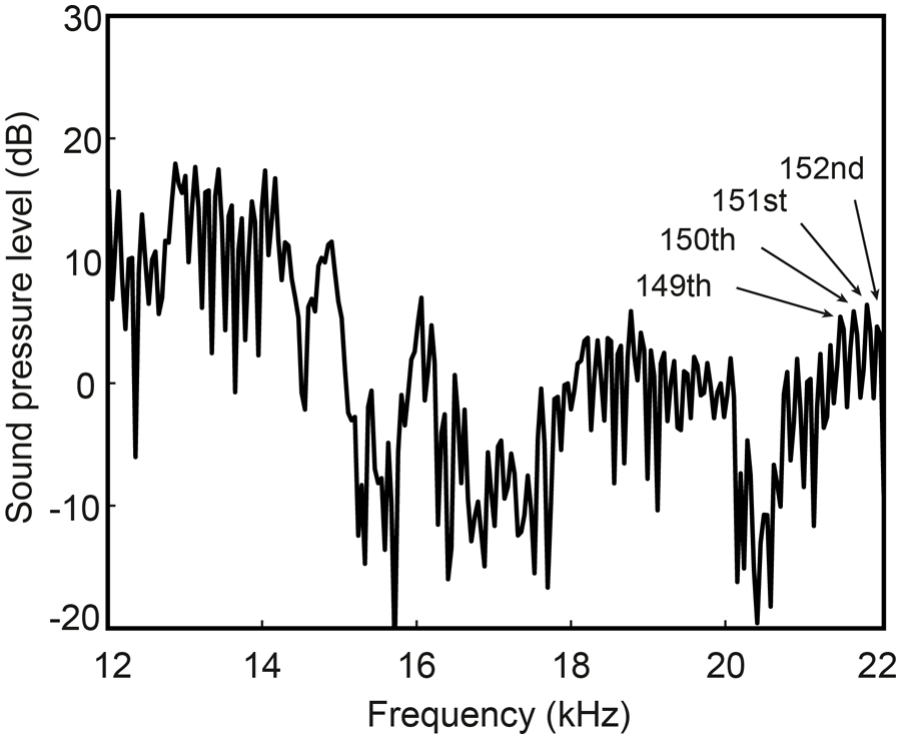
**High-frequency spectrum of the spoken vowel /e/ from one male subject (taken from the word “say”).** Harmonic energy is measurable beyond the 150th harmonic at nearly 22 kHz (indicated by labeled arrows).

Since temporal information is preserved in HFE and potentially useful for talker identification (Liss et al., 2010), it is possible that listeners had access to gender-specific information provided by speaking rate, although there are mixed findings on gender differences in speaking rate (e.g., Jacewicz et al., 2010; Clopper and Smiljanic, 2011). Carbonell et al. (2011) found that acoustic measures of rhythm could distinguish speaker gender somewhat, but the relevant information tended to lay in lower frequency bands. Another potential explanation for listeners’ success in gender discrimination is that listeners were able to extract fundamental frequency (F0) information from HFE sufficient to give rise to pitch perception. Listeners did report both perception of pitch and melody recognition (for the sung tokens). This implies that (1) harmonic energy was strong enough in level to preserve F0 information, and (2) listeners were extracting F0 from either the temporal fine structure of the signal, the envelope of the time waveform of the signal, or combination tones. It has generally been assumed that there is little to no harmonic energy above 6 kHz during voicing, until a recent report by Ternstrom (2008) of such harmonic energy in singing (see also Fry and Manen, 1957). Harmonic energy above 6 kHz has not been reported for speech, however. Examination of HFE in normal speech tokens here revealed harmonic energy beyond 6 kHz in many (but not all) subjects, and out to 20 kHz in rare cases (**Figure 3**).

*Post hoc* analysis offered additional explanation. The overall experimental error rate was 7.8% for gender discrimination and 0.7% for production mode discrimination. While listeners performed slightly better for speech stimuli than singing stimuli, this difference did not reach significance (*t* = 1.912, *p* = 0.069). There was no significant difference between performance for male and female voice stimuli (*t* = 1.642, *p* = 0.115). There was a difference between performance on the first 5 s of the SSB recordings and the last 5 s that was highly significant (*t* = 8.186, *p* < 0.001). This difference was reflected in the error rates for these stimuli, with the error rate for the last 5 s of the SSB recordings (12.9%) being more than three times greater than the error rate for the first 5 s stimuli (3.9%). This trend was consistent for both speech and singing.

Voiceless fricatives, particularly /s/ phonemes, were more prevalent in the first 5 s of SSB than the last 5 s. Since voiceless fricatives generally produce high amounts of HFE (Maniwa et al., 2009), it is possible that listeners were using gender differences in HFE found in voiceless fricatives to accomplish the gender discrimination task for speech and perhaps singing. This notion corroborates a report by Schwartz (1968) that listeners could discriminate the gender of speakers of isolated voiceless fricatives, although the stimuli used there were full bandwidth recordings. Previous research showing significant gender differences in HFE in voiceless fricatives (Jongman et al., 2000; Monson et al., 2012) suggests gender discrimination could be accomplished on this basis. However, overall performance with stimuli that contained *no* voiceless fricatives (i.e., last 5 s of singing) was still quite good (84.6% correct), suggesting other gender discrimination cues are available in the high frequencies.

Listener performance was analyzed using overall scores, gender discrimination scores, and production mode discrimination scores as the dependent variables in separate forward step-wise linear regression analyses with age, years of musical training, and the minimum pure tone thresholds (of the two ears) at each octave as possible predictors. None of these variables predicted overall performance at the α = 0.05 level. However, minimum threshold at 8 kHz did somewhat predict performance on the production mode discrimination task (β = –0.539, *p* = 0.008). Minimum pure tone threshold at 500 Hz was found to moderately predict performance on the gender discrimination task (β = –0.522, *p* = 0.011). There was no significant effect of listener gender on performance for either production mode (*t* = 1.428, *p* = 0.168) or gender discrimination (*t* = 0.205, *p* = 0.84).

The fact that nearly all listeners could identify the words being spoken was not predicted, particularly since they were not instructed to do so *a priori*. This result provides another example indicating the possibility of extracting speech intelligibility information from HFE (albeit with multiple repetitions). One alternative explanation is that listeners were first identifying the melody of the song being sung as SSB, and using this as a cue to identify the SSB lyrics being spoken. Three listeners who could not identify the spoken lyrics did identify the song. A counter example, however, was one listener that correctly identified the spoken lyrics but not the song being sung. To further examine some of these issues, four supplementary experiments were conducted.

## SUPPLEMENTARY EXPERIMENTS

### EXPERIMENT S1: ADDITION OF LOW-FREQUENCY MASKER

**FIGURE 4:**
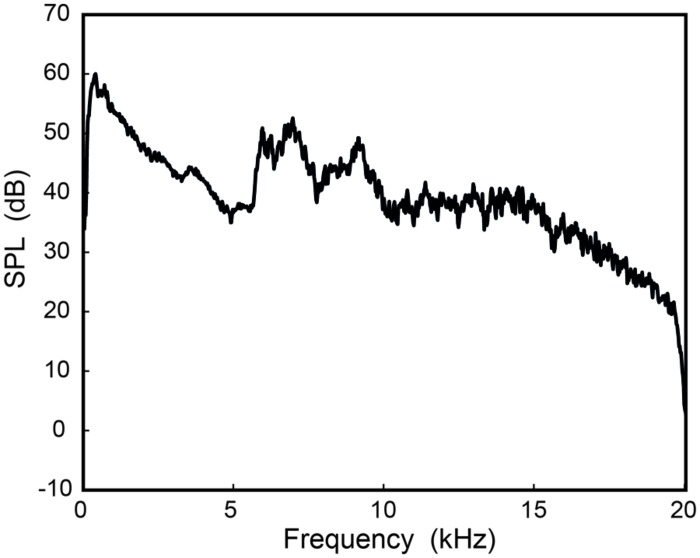
**An HFE stimulus (from **Figure 1**) with the addition of the speech-shaped noise low-frequency masker.** The masker was set to the same SPL as the original HFE stimulus. The spectrum was recorded at the position of the listener (1 m from the loudspeaker, on axis).

To check that listeners were not using combination tones to perform the task of gender discrimination and song identification, the main experiment was replicated with the addition of a low-frequency masking noise to the HFE stimuli. The masking noise was speech-shaped noise generated according to the ANSI (1992) standard, and then low-pass filtered at 5657 Hz using a 32-pole Butterworth filter. The level of the masker was set to be equal to the HFE presentation level (73 dB SPL at the ear), and summed with each SSB HFE stimulus used previously, resulting in each of the new 60 stimuli having an overall signal RMS level of 76 dB SPL. This level approximates the overall level of normal singing at this distance (Monson et al., 2012), but the level of the HFE relative to the low-frequency energy is markedly higher than would normally be the case (i.e., this condition represents a situation with amplified HFE). **Figure 4** shows the LTAS of one stimulus (from **Figure 1**) with the addition of the low-frequency masker, again recorded in the listening environment. Aside from this change in stimuli, the experimental procedure was identical to that for the main experiment.

Seven inexperienced listeners participated in the experiment (five female). Age ranged from 19 to 32 years, with a mean age of 23 years. All listeners except one had audiometric thresholds better than 15 dB HL in at least one ear for all frequencies up to 8 kHz. One listener had thresholds of 25 and 20 dB HL at 2 and 4 kHz, respectively. One listener had thresholds worse than 15 dB HL in both ears at 16 kHz.

All listeners were able to perform the discrimination tasks successfully (*p* < 0.0001). Mean scores were 99.5% correct for production mode discrimination and 91.4% correct for gender discrimination. All listeners correctly identified the song being sung as SSB. All listeners correctly identified the words being spoken as lyrics to SSB (see **Figure 2**).

### EXPERIMENT S2: PRESENTATION OF SPEECH ONLY

To eliminate the possibility of listeners using the identification of the sung melody as a cue for identifying the spoken lyrics, Experiment S1 was replicated using only the speech HFE stimuli. The Alvin program and instructions were modified to include only “Male Speech” and “Female Speech” as response choices, thereby removing any cues that the speech consisted of song lyrics or words of any familiarity. All experimental conditions were otherwise identical. This change resulted in a gender discrimination task with a total of 30 trials (15 speakers × 2 time segments). Seven inexperienced listeners participated in the experiment (five female). Age ranged from 18 to 27 years, with a mean age of 22 years. All listeners had audiometric thresholds better than 15 dB HL in at least one ear for all frequencies up to 8 kHz. Three listeners had thresholds worse than 15 dB HL in both ears at 16 kHz.

All listeners were able to perform the gender discrimination task successfully (*p* < 0.0005), with a mean score of 94.8% correct. All listeners except one correctly identified the words being spoken as lyrics to SSB.

### EXPERIMENT S3: ATTENTION TO PHONETIC CONTENT

There were varied responses from listeners when asked how soon they identified the song and spoken words. Some listeners claimed ability to recognize the speech on the first or second trial. For a more formal investigation of this possibility, three additional listeners with normal hearing participated in a modified version of Experiment S2 (speech only condition). These three listeners were asked after each trial to respond to the question “Did you understand what he/she said?” Response choices were “Yes,” “No,” or “Maybe (I think so, but I’m not sure).”

Results for the three listeners (L1, L2, L3) were: the first “Yes” circled was on Trial 2, Trial 12, and Trial 4 for listeners L1, L2, and L3, respectively; and the total number of “Yes” responses was 25/30, 17/30, and 27/30 for listeners L1, L2, and L3, respectively. This result indicates that some listeners can quickly identify words being spoken using only HFE. All three listeners correctly identified the words as lyrics to SSB following the experiment.

### EXPERIMENT S4: DECREASED LEVELS

To assess listeners’ abilities to perform the discrimination tasks at realistic HFE levels, Experiment S1 was replicated with the stimuli attenuated to an overall signal RMS level of 53 dB SPL (50 dB SPL HFE level, 50 dB SPL masker level). This HFE level is the average HFE level for normal singing (Monson et al., 2012), but, again, higher relative to low-frequency energy than would normally be the case. Stimuli were presented binaurally over calibrated Sennheiser HD 280 headphones. The experimental procedure was the same. Six inexperienced listeners with self-reported normal hearing participated in the experiment (four female). Age ranged from 24 to 30 years (mean = 27).

All listeners were able to perform the discrimination tasks successfully (*p* < 0.0001). Mean scores were 97.2% correct for production mode discrimination and 95.3% correct for gender discrimination. All listeners correctly identified the song being sung as SSB and the words being spoken as lyrics to SSB (see **Figure 2**).

### EXPERIMENT S5: SPEECH LEVELS

Average levels for normal speech are 62 dB SPL for low-frequency energy and 47 dB SPL for HFE (Monson et al., 2012). As a final test to simulate speech listening conditions, Experiment S4 was replicated with the low-frequency masker level increased and HFE level decreased to match these levels precisely. Five inexperienced listeners with self-reported normal hearing participated in the experiment (four female). Age ranged from 25 to 29 years (mean = 27).

All listeners were able to perform the discrimination tasks successfully (*p* < 0.01). Mean scores were 90.3% correct for production mode discrimination and 84% correct for gender discrimination. Three of the five listeners correctly identified both the song and spoken lyrics as SSB (see **Figure 2**).

## DISCUSSION

Gender discrimination and production mode discrimination performance was robust in the presence of low-frequency noise. As expected, performance in both tasks diminished in the most adverse (and most realistic) listening condition (Experiment S5). However, this condition represents a conservative estimate of performance in typical speech listening conditions since a constant-amplitude low-frequency noise masker was used whereas real speech is amplitude modulated, providing listeners the opportunity to “glimpse” the higher frequency bands during low amplitude troughs in the low frequencies (Cooke, 2006).

The successful identification of the lyrics by nearly every listener when presented with only speech HFE (Experiment S2) confirms that there is intelligibility information in HFE that is useful in the presence of low-frequency noise, although this information was presented several times by multiple talkers before listeners were asked to identify the speech. However, listeners were also given no instruction *a priori* to identify the speech. The significance of this result is that our stimuli were devoid of all low-frequency cues thought to be most important for speech intelligibility. While humans do show remarkable ability to decipher speech when spectral cues are severely degraded, demonstrations of this have typically included at least some representation of structure in low-frequency energy (Remez et al., 1981; Rosen et al., 1981; Shannon et al., 1995; Lippmann, 1996).

Listeners reported perception of pitch and melody recognition. It has been widely accepted that individual harmonics of a tone complex must be less than ∼5 kHz to perceive the missing fundamental (Ritsma, 1962), but a recent finding has contradicted this notion. Oxenham et al. (2011) used synthetic stimuli consisting of several equal-strength upper harmonics of fundamental frequencies (F0) ranging from 400 to 2000 Hz. Listeners were successful in pitch discrimination and pitch matching tasks even when all harmonics were above 5 kHz. The successful gender discrimination demonstrated here, if based on pitch cues, required discriminating typical female (∼200 Hz) and male (∼100 Hz) speaking F0s with harmonics at much lower levels in the speech stimuli. Melody recognition for the singing used here required perception of the missing fundamental with F0s ranging from 98 to 880 Hz. This study suggests the results from Oxenham et al. (2011) are relevant to naturally occurring stimuli, but it should be noted that their study involved partially resolved harmonics, whereas this study involved entirely unresolved harmonics.

Other HFE information was likely used in combination with pitch for these tasks, such as rhythm, loudness, and/or timbre cues that can help to identify recognizable melodies (White, 1960; McDermott et al., 2008), and potential gender differences in voiceless fricatives (Schwartz, 1968). We posit pitch as a likely cue because of listeners’ reports of pitch perception and because gender discrimination accuracy was maintained for tokens of singing with no voiceless fricatives. Notably, any perceptual cues obtained (temporal information, gender differences, pitch, phonetic content) were extracted solely from HFE in the presence of intense background noise at lower frequencies.

We questioned how much learning was necessary to accomplish these tasks. There was no significant difference between the number of errors made in the first half of the trials and second half of the trials (*t* = 1.27, *p* = 0.211). There was a significant difference between the number of extra repetitions (after initial presentation) required in the first half of trials and second half of trials (*t* = 2.98, *p* < 0.01), but the mean number of extra repetitions for each trial across listeners was less than 0.8 for all trials and less than 0.5 for 95% of the trials.

In conclusion, HFE provides perceptual information about a speech signal, including cues sufficient for gender discrimination. Since these cues are useful in the presence of a constant high-level low-frequency noise masker, HFE cues are likely useful (albeit perhaps redundant) in the presence of low-frequency speech energy. It is possible that HFE becomes of greater perceptual significance when low-frequency energy is disrupted or degraded, as is the case in noisy environments and in certain types of hearing loss (see also Berlin, 1982; Badri et al., 2011). Continued characterization of this portion of the speech spectrum will elucidate the cues provided by HFE, which should lead to better understanding of how to represent these cues for normal-hearing and hearing-impaired listeners.

## Conflict of Interest Statement

The authors declare that the research was conducted in the absence of any commercial or financial relationships that could be construed as a potential conflict of interest.
